# USING THE AGE-FRIENDLY INVENTORY AND CAMPUS CLIMATE SURVEY AT A MAJOR CANADIAN UNIVERSITY

**DOI:** 10.1093/geroni/igae098.1587

**Published:** 2024-12-31

**Authors:** Chantelle Zimmer, Lindsay Morrison, Maya Goerzen, David Hogan, Ann Toohey, Jennifer Hewson, Meghan McDonough, Gwen McGhan

**Affiliations:** University of Calgary, Calgary, Alberta, Canada; University of Calgary, Calgary, Alberta, Canada; University of Calgary, Calgary, Alberta, Canada; University of Calgary, Calgary, Alberta, Canada; University of Calgary, Calgary, Alberta, Canada; University of Calgary, Calgary, Alberta, Canada; University of Calgary, Calgary, Alberta, Canada; Faculty of Nursing, University of Calgary, Calgary, Alberta, Canada

## Abstract

Since the University of Calgary became a member of the AFU Global Network, establishing a benchmark of the institution’s age-friendliness has been a priority for the Brenda Strafford Centre on Aging. The Age-Friendly Inventory and Campus Climate Survey was selected for our baseline assessment because it captures both objective and subjective elements of age-friendliness that align with the AFU principles. Prior to data collection, the instrument was modified for our national and institutional context by adding, removing, and changing the language of some items. The Age-Friendly Inventory was completed by administrators to determine the current status of our campus practices and environmental features, while the Campus Climate Survey was completed by faculty, staff, and students to understand their awareness and perceptions of these practices and features. A total of 10 administrators, 178 faculty, 608 staff, and 1167 students participated in the assessment. The results indicated that our university is moderately age-friendly, but participants were generally unaware of its age-friendly elements. To generate action items from the results, we analyzed the data further. Each inventory and survey item was mapped onto the AFU principle it most closely aligned with and then grouped with similar items to form categories for each principle. Through this process we identified areas of strength and growth for each principle, and prioritized principles (1, 4, 6, and 10) for our Centre to address. The findings from this study will inform an action plan to raise awareness of and enhance the University of Calgary’s age-friendliness.

